# Palliative care providers’ use of digital health and perspectives on technological innovation: a national study

**DOI:** 10.1186/s12904-021-00822-2

**Published:** 2021-08-07

**Authors:** Jason Mills, Jennifer Fox, Raechel Damarell, Jennifer Tieman, Patsy Yates

**Affiliations:** 1 Centre for Healthcare Transformation, Queensland University of Technology, Kelvin Grove, QLD 4061 Australia; 2 College of Nursing and Health Sciences, Flinders University, Adelaide, Australia

**Keywords:** Digital health, Innovation, Palliative care, Survey, Technology

## Abstract

**Background:**

While the need for digital health capability and technological innovation in palliative care services is growing rapidly, relatively little is known about the current uptake and views of individual palliative care practitioners. This study aims to explore palliative care practitioners’ current use of and perspectives on digital health innovation in palliative care.

**Methods:**

A descriptive cross-sectional survey with a web-based questionnaire was used. Participants were multidisciplinary palliative care practitioners in Australia.

**Results:**

Surveys were returned by 170 medical, nursing, and allied health practitioners working in palliative care. Most respondents reported using a variety of digital health technology associated with clinical information systems, mobile devices, SMS text messaging, teleconferencing, and Wi-Fi. These technologies were used for the purpose of communicating with other health professionals, accessing web-based or mobile health palliative care resources, collecting or managing patient data, and providing information or education. However, few reported electronic access to patients’ advance care planning documentation or could update these data. Respondents were moderately confident in their ability to use digital health, held positive beliefs that palliative care could be enhanced through digital health, and were generally supportive of ongoing innovation through digitally-enable models of care. Palliative care providers would most like to see digital health innovations in the areas of client health records, telehealth, and personal health tracking.

**Conclusion:**

This is the first national study of digital health in Australian palliative care providers. It contributes new knowledge in this important area of palliative care practice to guide policy and education, whilst informing future directions for research.

## Introduction

The combined impacts of population ageing, and emerging global pandemics raise growing concerns about the provision of palliative care, with pressing implications for ongoing innovation and greater use of technology [1, 2]. As increasingly discussed in contemporary palliative care discourse, the nascent field of digital health represents an important area with potential to challenge and support advances in policy, practice and research [3, 4].

Indeed, digital health can support the strengthening and scaling up of palliative care worldwide.^5^ In its *Global Strategy on Digital health*, the World Health Organisation (WHO) defines digital health as ‘the field of knowledge and practice associated with the development and use of digital technologies to improve health’ [5]. Digital health widens the scope of earlier fields (e.g., eHealth) to encompass a broader range of smart or connected wearable devices, along with other technologies that may involve the use of robotics, big data analytics or applications of artificial intelligence such as machine learning [5]. It also extends focus towards the empowerment and agency of health professionals and healthcare consumers living in an increasingly digital society; taken together, digital health reflects an expansion and cultural transformation of traditional healthcare [6].

Situating palliative care within this larger scale perspective is becoming more important, given the international trend towards digital health interventions that offer digitally enabled models of care for digital consumers to progress towards a more equitable state of universal health coverage [5]. To this end, many countries, including Australia and across the Americas, have adopted the use of electronic health records and established national digital health strategies to advance innovation and clinical outcomes [7, 8]. National eHealth policies are in place across sub-Saharan Africa, and digital health approaches are being developed to improve access to and enhance delivery of palliative cancer care in Nigeria, Uganda and Zimbabwe [9]. Given the importance of timely and reliable access to health records in a variety of contexts including carer support, advance care planning and associated health directives for end-of-life care, there is a pressing need to better understand palliative care within this new digital context.

In the palliative care literature to date, studies have largely focused on the feasibility and impact of specific modes or categories of technology used - including information and communication technologies (ICT), eHealth, mHealth, and telehealth [10–14]. However, there is scant research into the nexus of palliative care and the contemporary landscape of digital health. While tangible benefits can be realised through investigation of key stakeholder perspectives [9], we found no such studies of individuals providing palliative care. Indeed, it appears that very little is known about the use of, and perspectives on, digital health by individual palliative care providers. Therefore, the aim of this study was to explore palliative care providers’ current use of digital health and their perspectives on technological innovation.

## Methods

A descriptive cross-sectional design was used to conduct a national survey of Australian medical, nursing, and allied health professionals working in palliative care. The anonymous survey was web-based, with data questionnaire development and data collection carried out using the widely used Research Electronic Data Capture (REDCap) platform [15]. The development of a structured questionnaire was informed by a review of the extant literature, with feedback from reviewers outside the research team used to guide content validity. This approach to questionnaire design and piloting is consistent with the survey research methods outlined by Addington-Hall [16]. Reviewers were identified through their expertise in the subject matter areas of palliative care and digital health, with some holding postgraduate qualifications in eHealth and Fellowship of the Australasian College of Health Informatics. The only substantive amendment was the addition of WHO’s definition of digital health for the purpose of ensuring a standardised understanding of the term ‘digital health’ for survey respondents [6].

The 20-item questionnaire included a combination of Likert and multiple-choice questions to capture data relating to individual demographics and electronic access to advance care planning records, as well as the uptake and purpose of specific digital health technologies used by providers of palliative care. A series of agree-disagree statements were included for respondents to rate the extent to which they agreed or disagreed with statements relating to their use of digital health in the areas of confidence, benefits, concerns, resources and training. Finally, respondents were asked to complete (free text) the statement ‘If there was one digital health innovation I would love to see in palliative care, it would be … ’.

Eligible participants were healthcare professionals registered to practise in Australia and currently providing palliative care in a general or specialist palliative care setting. Given the lack of any national register to quantify or provide access to all palliative care practitioners in Australia, medical, nursing, and allied health professionals were primarily recruited via electronic survey invitations distributed by their respective national palliative care associations (Australian and New Zealand Society of Palliative Medicine, Australian Allied Health in Palliative Care, and Palliative Care Nurses Australia). Members of Australian Allied Health in Palliative Care included dieticians, physiotherapists, psychologists, social workers, speech pathologists, and occupational therapists. These three associations reported survey distribution to a combined membership of almost 1000 at the time of the survey. The survey link was further disseminated via social media platforms. The survey went live mid-November 2018 and remained open until early January 2019.

All quantitative survey data were exported from REDCap directly to IBM SPSS 25, with descriptive and inferential statistics analysed with statistical significance set at *p* ≥ 0.05. Survey responses with less than 80% of the questionnaire completed were excluded from analysis. Qualitative data were exported directly to Microsoft Excel and cross-tabulated for qualitative content analysis [17]. Two members of the research team carried out deductive content analysis independently and reached agreement, using the *WHO Classification of Digital Health Interventions* (classification groupings 1.0 *Clients* and 2.0 *Healthcare providers*) as an analytical frame to categorise the digital health innovations described by respondents [18]. While conceptual findings are interpreted from data through inductive content analysis, deductive content analysis is more appropriate when the structure of analysis is operationalised within the context of previous knowledge [17]. In this way, the process of deductive content analysis enabled a direct mapping of the response data to the existing classification framework developed by WHO [18].

The study received approval from the human research ethics committees at Queensland University of Technology (Ref 1,800,000,954, 30/10/18) and Flinders University (Ref.OH-00196, 12/11/18), Australia. Informed consent to participate was implied through voluntary engagement with the survey, after receiving a research participant invitation together with information about the study.

## Results

One hundred-seventy survey responses were received from palliative care providers across metropolitan, regional/rural and remote areas of Australia, with palliative care doctors, nurses and allied health professionals from each State and Territory represented.

### Non-users of digital health technologies

Five palliative care providers indicated that they did not use digital health technologies. Non-user respondents were female (100%), from a range of ages (18–29 years *n* = 1, 30–39 years *n* = 2, 50–59 years *n* = 1, 60+ years *n* = 1), within metropolitan (*n* = 3) or regional (*n* = 2) areas. Two respondents were allied health professionals and three were nurses. Most had less than 10 years’ experience working in palliative care (1–5 years *n* = 2, 6–10 years *n* = 2, > 10 years *n* = 1).

### Users of digital health technologies

One hundred and fifty-four respondents reported using digital health technology or applications in their provision of palliative care. The majority were female (83.8%), aged ≥40 years (72%), nurses (41.6%) and had > 10 years’ experience (52.6%). Most were located in New South Wales (29.2%), Queensland (28.6%) or Victoria (19.5%) and served metropolitan (64.3%) areas. (see Table 1).

**Table 1:** Characteristics of palliative care providers who use digital technology

Characteristic	All respondents (***N*** = 154)		Metropolitan (***N*** = 99)		Regional (***N*** = 49)		Remote (***N*** = 6)		Allied health (***N*** = 36)		Medical (***N*** = 54)		Nursing (***N*** = 64)	
	N	%	N	%	N	%	N	%	N	%	N	%	N	%
**Sex**														
Female	129	83.8	82	82.8	41	83.7	6	100.0	33	91.7	34	63.0	62	96.9
Male	24	15.6	17	17.2	7	14.3	–	–	2	5.6	20	37.0	2	3.1
Other	1	0.6	–	–	1	2.0	–	–	1	2.8	–	–	–	–
**Age**														
18–29 years	9	5.8	7	7.1	1	2.0	1	16.7	5	13.9	2	3.7	2	3.1
30–39 years	34	22.1	28	28.3	6	12.2	–	–	11	30.6	16	29.6	7	10.9
40–49 years	43	27.9	26	26.3	15	30.6	2	33.3	6	16.7	19	35.2	18	28.1
50–59 years	47	30.5	28	28.3	18	36.7	1	16.7	12	33.3	11	20.4	24	37.5
60+ years	21	13.6	10	10.1	9	18.4	2	33.3	2	5.6	6	11.1	13	20.3
**Location**														
Australian Capital Territory	4	2.6	4	4.0	–	–	–	–	–	–	2	3.7	2	3.1
New South Wales	45	29.2	25	25.3	19	38.8	1	16.7	8	22.2	13	24.1	24	37.5
Northern Territory	4	2.6	1	1.0	1	2.0	2	33.3	2	5.6	–	–	2	3.1
Queensland	44	28.6	29	29.3	14	28.6	1	16.7	14	38.9	10	18.5	20	31.3
South Australia	5	3.2	5	5.1	–	–	–	–	3	8.3	1	1.9	1	1.6
Tasmania	5	3.2	2	2.0	3	6.1	–	–	–	–	3	5.6	2	3.1
Victoria	30	19.5	18	18.2	12	24.5	–	–	7	19.4	15	27.8	8	12.5
Western Australia	17	11.0	15	15.2	–	–	2	33.3	2	5.6	10	18.5	5	7.8
**Area**														
Metropolitan	99	64.3	–	–	–	–	–	–	25	69.4	42	77.8	32	50.0
Regional	49	31.8	–	–	–	–	–	–	11	30.6	11	20.4	27	42.2
Remote	6	3.9	–	–	–	–	–	–	–	–	1	1.9	5	7.8
**Profession**														
Allied health	36	23.4	25	25.3	11	22.4	–	–	–	–	–	–	–	–
Medical	54	35.1	42	42.4	11	22.4	1	16.7	–	–	–	–	–	–
Nursing	64	41.6	32	32.3	27	55.1	5	83.3	–	–	–	–	–	–
**Years working in palliative care**														
1–5 years	4	58.6	33	33.3	9	18.4	2	33.3	11	30.6	20	37.0	13	20.3
6–10 years	29	18.8	19	19.2	7	14.3	3	50.0	10	27.8	7	13.0	12	18.8
> 10 years	81	52.6	47	47.5	33	67.3	1	16.7	15	41.7	27	50.0	39	60.9

### Access to advance care planning documentation and digital health technologies/resources used in palliative care

Few respondents reported access to patients’ advance care planning data in the My Health Record system (*n* = 20, 13%), or the ability to update these data (*n* = 8) (Table 2). Other than commonly used ICT hardware and electronic mail applications, the digital technologies mostly used by palliative care providers were clinical information systems (73.4%), mobile devices (66.2%), SMS text messaging (63.6%), teleconferencing (55.8%), and Wi-Fi (55.2%) (see Table 2). Digital health technologies or applications were most often used for the purpose of communicating with other health professionals (87.0%), accessing web-based or mobile health palliative care resources (76.0%), collecting or managing patient data (70.1%), and providing information (66.2%) or education (64.9%) (Table 3).

**Table 2:** Palliative care provider use of digital health technologies, applications and resources

	All respondents (***N*** = 154)		Metropolitan (***N*** = 99)		Regional (***N*** = 49)		Remote (***N*** = 6)		Allied health (***N*** = 36)		Medicine (***N*** = 54)		Nursing (***N*** = 64)	
Service use and technologies used	N	%	N	%	N	%	N	%	N	%	N	%	N	%
**Health professionals have access to patients’ advance care planning data in the MyHealth Record system**														
Yes	20	13.0	10	10.1	6	12.2	4	66.7	4	11.1	8	14.8	8	12.5
No	103	66.9	68	68.7	33	67.3	2	33.3	22	61.1	37	68.5	44	68.8
Unsure	31	20.1	21	21.2	10	20.4	–	–	10	27.8	9	16.7	12	18.8
**Health professionals can update patient data in the MyHealth Record System**														
Yes	8	40.0	4	40.0	1	16.7	3	75.0	1	25.0	2	25.0	5	62.5
No	2	10.0	–	–	1	16.7	1	25.0	1	25.0	–	–	1	12.5
Unsure	10	50.0	6	60.0	4	66.7	–	–	2	50.0	6	75.0	2	25.0
Missing	134	–	89	–	43	–	2	–	32	–	46	–	56	–
**Digital technologies or applications used**														
Desktop or laptop computer	151	98.1	98	99.0	47	95.9	6	100.0	34	94.4	54	100.0	63	98.4
Email	150	97.4	95	96.0	49	100.0	6	100.0	35	97.2	54	100.0	61	95.3
Telephone/Fax (VoIP)	74	48.1	41	41.4	28	57.1	5	83.3	22	61.1	16	29.6	36	56.3
SMS text messaging	98	63.6	56	56.6	38	77.6	4	66.7	18	50.0	40	74.1	40	62.5
Clinical information system (Patient records/ documentation e.g., PalCare)	113	73.4	73	73.7	36	73.5	4	66.7	24	66.7	43	79.6	46	71.9
Teleconferencing	86	55.8	45	45.5	36	73.5	5	83.3	17	47.2	28	51.9	41	64.1
Videoconferencing	71	46.1	36	36.4	30	61.2	5	83.3	19	52.8	21	38.9	31	48.4
Mobile device (smartphone, tablet, wearable)	102	66.2	65	65.7	34	69.4	3	50.0	20	55.6	43	79.6	39	60.9
Social media (e.g., Facebook or Twitter)	31	20.1	22	22.2	9	18.4	–	–	7	19.4	11	20.4	13	20.3
Blog	10	6.5	7	7.1	3	6.1	–	–	2	5.6	4	7.6	4	6.3
Remote monitoring	1	0.6	1	1.0	–	–	–	–	–	–	1	1.9	–	–
Website browser	69	44.8	47	47.5	20	40.8	2	33.3	14	38.9	32	59.3	23	35.9
Wireless internet (Wi-Fi)	85	55.2	50	50.5	32	65.3	3	50.0	15	41.7	37	68.5	33	51.6
Wired internet	47	30.5	32	32.3	14	28.6	1	16.7	14	38.9	17	31.5	16	25.0
Health/PC apps	68	44.2	41	41.4	24	49.0	3	50.0	6	16.7	29	53.7	33	51.6
DVD player	17	11.0	9	9.1	7	14.3	1	16.7	4	11.1	3	5.6	10	15.6
Digital audio recording device	9	5.8	3	3.0	6	12.2	–	–	1	2.8	3	5.6	5	7.8
Virtual reality device	3	1.9	1	1.0	2	4.1	–	–	1	2.8	–	–	2	3.1

*CD* Compact Disc, *DVD* Digital Video Disc, *PC* Palliative care, *SMS* Short message service, *VoIP* Voice over Internet Protocol

**Table 3:** Purpose of use of digital health technologies / applications

	All respondents (***N*** = 154)		Metropolitan (***N*** = 99)		Regional (***N*** = 49)		Remote (***N*** = 6)		Allied health (***N*** = 36)		Medicine (***N*** = 54)		Nursing (***N*** = 64)	
Purpose of use	N	%	N	%	N	%	N	%	N	%	N	%	N	%
Access to web-based or mobile health PC resources	117	76.0	71	71.7	40	81.6	6	100.0	27	75.0	45	83.3	45	70.3
Telehealth consultation	47	30.5	19	19.2	23	46.9	5	83.3	8	22.2	15	27.8	24	37.5
Communication with patients/family	82	53.2	43	43.4	34	69.4	5	83.3	23	63.9	20	37.0	39	60.9
Communication with other health professionals	134	87.0	83	83.8	45	91.8	6	100.0	33	91.7	48	88.9	53	82.8
Collect or manage patient data	108	70.1	65	65.7	38	77.6	5	83.3	25	69.4	40	74.1	43	67.2
Provide information	102	66.2	64	64.6	33	67.3	5	83.3	25	69.4	30	55.6	47	73.4
Provide education	100	64.9	57	57.6	38	77.6	5	83.3	24	66.7	29	53.7	47	73.4
Decision-making aid	59	38.3	31	31.3	23	46.9	5	83.3	12	33.3	18	33.3	29	45.3
Promote advance care planning	45	29.2	25	25.3	16	32.7	4	66.7	5	13.9	14	25.9	26	40.6
Support symptom management	77	50.0	47	47.5	24	49.0	6	100.0	11	30.6	32	59.3	34	53.1
Support patient recreation/relaxation	26	16.9	13	13.1	10	20.4	3	50.0	12	33.3	6	11.1	8	12.5
Other	7	4.5	4	4.0	3	6.1	–	–	6	16.7	–	–	1	1.6

*PC* Palliative Care

Chi-square analyses with Fisher’s exact 2-sided test for significance (*p* ≤ 0.05) were used to examine potential differences in technologies used and purpose of use based on area (metropolitan vs. regional only given small cell sizes for the remote category). These comparisons showed that a significantly greater proportion of regional providers, compared to metropolitan providers, used SMS text messaging, χ2(1) = 6.23, *p* = 0.018, teleconferencing, χ2(1) = 10.38, *p* = 0.002, and videoconferencing, χ2(1) = 8.20, *p* = 0.05.

Significantly more regional palliative care providers, compared to metropolitan providers, used digital health technologies for telehealth consultations, χ2(1) = 12.42, *p* = 0.001, to communicate with patients/family, χ2(1) = 8.85, *p* = 0.003, and provide education, χ2(1) = 5.69, *p* = 0.019.

Chi-square analyses, with 2-sided significance (*p* ≤ 0.05), were conducted to explore potential differences in technology use and purpose between professions (allied health, medicine, nursing). More palliative care providers from the medical and nursing profession, compared to allied health professionals, used mobile devices (smartphone, table, wearable), χ2(2) = 6.97, *p* = 0.031, websites, χ2(2) = 7.11, *p* = 0.029, Wi-Fi, χ2(2) = 6.88 *p* = 0.032, and health/palliative care apps, χ2(2) = 14.45, *p* = 0.001. Fewer medical professionals, compared to allied health and nursing, used digital technologies to communicate with patients/family, χ2(2) = 8.86, *p* = 0.012. A significantly greater proportion of nurses, compared to allied health professionals, used digital technologies to promote advance care planning, χ2(2) = 8.40, *p* = 0.015. A significantly greater proportion of allied health professionals, compared to medical and nursing professionals used these technologies to support patient recreation/relaxation, χ2(2) = 9.10, *p* = 0.011.

### Palliative care provider attitudes toward digital health technologies

Tables 4 and 5 report descriptive data for each of the attitudes toward digital health technology items based on area and profession. On average, palliative care providers were moderately confident in their ability to use digital health, held positive beliefs that palliative care could be enhanced through digital health, and were supportive of ongoing innovation through digitally-enable models of care. Overall, respondents were neutral in their concerns about safety and confidentiality of patient information, as well as their perceptions about having access to sufficient technical help to support the use of digital health technologies. Provider responses indicated moderate agreement that they would like access to training or educational resources to support their use of digital health in palliative care.

**Table 4:** Perceived service enhancement and perceived adequacy of technological capability or innovation by area

	All respondents (***N*** = 154)			Metro (***N*** = 99)			Regional (***N*** = 49)			Remote (***N*** = 6)		
Item	Mean (SD)	Min-Max	N	Mean (SD)	Min-Max	N	Mean (SD)	Min-Max	N	Mean (SD)	Min-Max	N
Confident utilising digital health to provide PC	3.72 (0.89)	1.00–5.00	148	3.70 (0.87)	1.00–5.00	94	3.81 (0.87)	1.00–5.00	48	3.17 (1.33)	1.00–4.00	6
Missing			6			5			1			–
PC can be enhanced through digital health	4.21 (0.69)	1.00–5.00	145	4.27 (0.63)	3.00–5.00	92	4.17 (0.78)	1.00–5.00	48	3.60 (0.55)	3.00–4.00	5
Missing			9			7			1			1
Supportive of ongoing innovation through digitally enabled models of care that improve quality and access to PC	4.27 (0.75)	1.00–5.00	149	4.38 (0.57)	3.00–5.00	95	4.13 (0.89)	1.00–5.00	48	3.67 (1.56)	1.00–5.00	6
Missing			5			4			1			–
Concerned about the safety and confidentiality of patient information in digital health	3.15 (1.01)	1.00–5.00	148	3.16 (0.98)	1.00–5.00	95	3.15 (0.98)	1.00–5.00	47	3.00 (1.67)	1.00–5.00	6
Missing			6			4			2			–
Do not have access to sufficient technical help to support me using digital health applications	2.91 (1.03)	1.00–5.00	148	2.95 (1.05)	1.00–5.00	94	2.79 (0.90)	1.00–5.00	48	3.33 (1.63)	1.00–5.00	6
Missing			6			5			1			–
Would like access to training and/or educational resources to support my use of digital health in PC	3.88 (0.77)	1.00–5.00	147	3.88 (0.78)	1.00–5.00	93	3.90 (0.66)	2.00–5.00	48	3.83 (1.47)	1.00–5.00	6
Missing			7			6			1			–

*PC* Palliative Care

**Table 5:** Perceived service enhancement and perceived adequacy of technological capability or innovation by profession

	Allied health (***N*** = 36)			Medicine (***N*** = 54)			Nursing (***N*** = 64)		
Item	Mean (SD)	Min-Max	N	Mean (SD)	Min-Max	N	Mean (SD)	Min-Max	N
Confident utilising digital health to provide PC	3.58 (0.91)	1.00–5.00	36	3.79 (0.78)	2.00–5.00	52	3.73 (0.97)	1.00–5.00	60
Missing			–			2			4
PC can be enhanced through digital health	4.19 (0.75)	2.00–5.00	36	4.27 (0.57)	3.00–5.00	51	4.17 (0.75)	1.00–5.00	58
Missing			–			3			6
Supportive of ongoing innovation through digitally enabled models of care that improve quality and access to PC	4.39 (0.55)	3.00–5.00	36	4.31 (0.54)	3.00–5.00	52	4.16 (0.97)	1.00–5.00	61
Missing			–			2			3
Concerned about the safety and confidentiality of patient information in digital health	2.89 (0.93)	1.00–5.00	35	3.33 (0.92)	2.00–5.00	52	3.15 (1.09)	1.00–5.00	61
Missing			1			2			3
Do not have access to sufficient technical help to support me using digital health applications	3.20 (0.96)	2.00–5.00	35	2.94 (1.07)	1.00–5.00	52	2.72 (1.00)	1.00–5.00	61
Missing			1			2			3
Would like access to training and/or educational resources to support my use of digital health in PC	4.06 (0.67)	2.00–5.00	36	3.80 (0.66)	2.00–5.00	51	3.85 (0.90)	1.00–5.00	60
Missing			–			3			4

*PC* Palliative Care

One-way ANOVAs (*p* ≤ 0.05), with Tukey post-hoc comparisons for significant results, were used to explore potential differences in confidence, enhancement, support of ongoing innovation, concern about safety and confidentiality, access and training needs based on area (metropolitan vs. regional) or profession (allied health, medicine, nursing). There was a statistically significant difference in support for ongoing innovation through digitally enabled models of care based on area, F (2,146) = 4.00, *p* = 0.02. A Tukey post-hoc test revealed that palliative care providers in metropolitan areas reported higher (approaching significance) support for ongoing innovation (*p* = 0.059) compared to those in remote areas. There was no statistically significant difference between the support of providers in metropolitan and regional areas, or by profession.

### Digital health innovations providers would like to see in palliative care

Most respondents reported they would like to see digital health innovations in the areas of client health records (*n* = 37); telemedicine (*n* = 22), and personal health tracking (*n* = 15). Table 6. reports the digital health innovations that respondents would like to see in palliative care, as classified by the WHO categories of digital health interventions.^16^ While a small number of responses did not directly map to these, they reflected a desire for improved connectivity through wireless infrastructure to access and enable technology for patient communication with families. For example, in response to the free-text question ‘If there was one digital health innovation I would love to see in palliative care, it would be … ’, one participant responded:

**Table 6:** Categories of digital health innovations providers would like to see in palliative care

WHO Digital Health Intervention Classification Category	n	%
1.4: Personal health tracking	15	14.2
1.6: On demand information services to client	8	7.5
2.2: Client health records	37	34.9
2.3: Healthcare provider decision support	3	2.8
2.4: Telemedicine	22	20.8
2.5: Healthcare provider communication	3	2.8
2.6: Referral coordination	1	0.9
2.8: Healthcare provider training	6	5.7
2.9: Prescription and medication management	3	2.8
Sundries (not classified elsewhere)	8	7.5

Simple - Wi-Fi for patients and families in inpatient palliative care unit to enhance communication options when patients are debilitated and family live away.

## Discussion

This national study explored Australian palliative care providers’ current use of digital health and their perspectives on technological innovation. Given that implementation of digital health interventions represents a cultural transformation of traditional healthcare [6], understanding the confidence and attitudes of healthcare professionals regarding the use of digital health technologies is a growing priority [19]. This research advances knowledge in that area, with scant research previously conducted in the context of palliative care providers.

In the present study, there was a large uptake of digital health by multi-disciplinary palliative care providers who were, on average, moderately confident in their ability to use digital health and held positive beliefs that palliative care could be enhanced through digital health. They were generally supportive of ongoing innovation through digitally-enable models of care. This supports earlier research that found rural nurses were able to accept, use, and report benefits of telehealth in paediatric hospice care, whilst cautioning about the importance of maintaining human connection [20]. The introduction of digitally enabled models of care presents both benefits and challenges for providers of palliative care [12, 21].

The use and purpose of digital health technologies reported by palliative care providers is largely consistent with those featured in emerging the research literature, except for scale of virtual reality use and notable omissions in the more advanced areas of robotics, big data, and artificial intelligence [22, 23]. Although, this is perhaps not surprising, given their early stage of development and implementation in palliative care. A diverse range of current and emerging technologies is evident in recent studies, with the use of electronic health records, virtual reality, mobile apps and wearable devices, or telehealth most commonly used by either specialist or non-specialist palliative care providers [24–32].

In the clinical context of the COVID-19 pandemic, there has been particular interest in the use of technology as part of the palliative care response. For instance, mobile devices to facilitate spiritual support for patients and video visits to enhance their communication with families [33]. Real-time global communication and a variety of online resources have been mobilised through virtual communities of practice using various digital platforms such as webinar, blog or social media (see for example, Twitter *#pallicovid*) [34]. The rapid implementation of telehealth consultations has been reported as both feasible and cost-effective, particularly in the case of social distancing restrictions and resource supply limitations in personal protective equipment [35, 36]. However, telehealth does not represent a panacea to supplant face to face palliative care consultations, and more rigorous evaluation is required [10].

A number of findings from this study were surprising. Of some concern, is that some respondents reported no access to Wi-Fi, signally limited communication options for patients and their families, and suggesting a poor level of digital health infrastructure. To provide quality palliative care, clinicians increasingly rely on adequate digital health infrastructure and support [35, 37]. Overall, there was moderate agreement among palliative care providers that they would like access to training or educational resources to support their use of digital health in palliative care. Given the broad scope of digital health, as it was defined for participants, it was surprising then, that a small number of respondents across a range of age groups reported not using digital health in their provision of palliative care. This raises important implications for the investigation of digital literacy in palliative care providers, as well as the provision of appropriate education and training to build workforce capacity for digital health [38]. Studies could usefully examine these factors in future research.

Under the Australian Government’s National Palliative Care Strategy, all healthcare professionals providing palliative care can play an important role in the promotion of advance care planning [39]. Interestingly, in this study nurses were more likely to use digital technologies to promote advance care planning in comparison to allied health professionals. Whilst workforce competencies of those engaged in advance care planning discussions have been highlighted as a potential issue [40], this finding may reflect a greater level of nurses’ access to digital technologies for this purpose. This will be important to monitor into the future, given the increasing investment in digital infrastructure and national guidelines to support advance care planning through the My Health Record (an online summary of key health information previously called eHealth record) [41].

Electronic access to advance care planning documentation was also a significant concern in this study, with only 13% of respondents reporting access to documentation about patient preferences through the My Health Record system. Although in May 2020, approximately 90% of the Australian population had a My Health record with the ability to store important documentation such as advance care plans and make these nationally accessible to healthcare professionals [42], this finding should be understood within the context of a generally low population prevalence of advance care planning uptake at the time of the survey, and an even lower rate of advance care directives being documented on My Health records [43]. Nonetheless, ongoing monitoring and improvement in electronic access to advance care planning documentation is warranted.

Given the respondents’ indications of need for innovation – and consistent with the WHO classification on digital health interventions – future research and development should consider prioritising digital health interventions in the areas of client health records, telehealth, and personal health tracking [18]. In addition, study surveys were distributed prior to Covid-19, thus this study provides a solid platform for a follow-up study to explore changes following Covid-19.

A limitation of this study is the convenience sampling method and inability to determine an accurate response rate due to survey distribution via social media, in addition to national palliative care associations. This limitation is associated with the absence of a national register of palliative care practitioners in Australia. It is possible that data from survey respondents may not reflect those who did not participate in the study. Given the survey was distributed via electronic mail and social media, there is potential for bias if only those with access to digital technology completed the survey. Additionally, because the cross-sectional study design captures data at a single snapshot in time, it cannot account for any changes in study variables that occur after the survey period. Despite these limitations, the strengths of this research include the participation of palliative care providers from each State and Territory; the sample represented the relatively small number of practitioners working in palliative care, as well as the diversity of workforce demographics, and clinical settings across metropolitan, regional/rural, or remote geographical areas of Australia.

In conclusion, this is the first national study of digital health in Australian palliative care providers. This work contributes new knowledge in this important area of palliative care practice to guide policy and education, whilst informing future directions for research.

## Data Availability

The data sets used and analysed during the current study are available from the corresponding author on reasonable request.
